# HIV-1 coreceptor usage prediction without multiple alignments: an application of string kernels

**DOI:** 10.1186/1742-4690-5-110

**Published:** 2008-12-04

**Authors:** Sébastien Boisvert, Mario Marchand, François Laviolette, Jacques Corbeil

**Affiliations:** 1 Centre de recherche du centre hospitalier de l'Université Laval, Québec (QC), Canada; 2 Département d'informatique et de génie logiciel, Université Laval, Québec (QC), Canada

## Abstract

**Background:**

Human immunodeficiency virus type 1 (HIV-1) infects cells by means of ligand-receptor interactions. This lentivirus uses the CD4 receptor in conjunction with a chemokine coreceptor, either CXCR4 or CCR5, to enter a target cell. HIV-1 is characterized by high sequence variability. Nonetheless, within this extensive variability, certain features must be conserved to define functions and phenotypes. The determination of coreceptor usage of HIV-1, from its protein envelope sequence, falls into a well-studied machine learning problem known as *classification*. The support vector machine (SVM), with string kernels, has proven to be very efficient for dealing with a wide class of classification problems ranging from text categorization to protein homology detection. In this paper, we investigate how the SVM can predict HIV-1 coreceptor usage when it is equipped with an appropriate string kernel.

**Results:**

Three string kernels were compared. Accuracies of 96.35% (CCR5) 94.80% (CXCR4) and 95.15% (CCR5 and CXCR4) were achieved with the SVM equipped with the *distant segments kernel *on a test set of 1425 examples with a classifier built on a training set of 1425 examples. Our datasets are built with Los Alamos National Laboratory HIV Databases sequences. A web server is available at .

**Conclusion:**

We examined string kernels that have been used successfully for protein homology detection and propose a new one that we call the *distant segments kernel*. We also show how to extract the most relevant features for HIV-1 coreceptor usage. The SVM with the *distant segments kernel *is currently the best method described.

## Background

The HIV-1 genome contains 9 genes. One of the genes, the *env *gene, codes for 2 envelope proteins named gp41 and gp120. The gp120 envelope protein must bind to a CD4 receptor and a coreceptor prior to cell infection by HIV-1. Two coreceptors can be used by HIV-1: the CCR5 (chemokine receptor 5) and the CXCR4 (chemokine receptor 4). Some viruses are only capable of using the CCR5 coreceptor. Other viruses can only use the CXCR4 coreceptor. Finally, some HIV-1 viruses are capable of using both of these coreceptors. The pathology of a strain of HIV-1 is partly a function of the coreceptor usage [1]. The faster CD4+ cell depletion caused by CXCR4-using viruses [2] makes the accurate prediction of coreceptor usage medically warranted. Specific regions of the HIV-1 external envelope protein, named hypervariable regions, contribute to the turnover of variants from a phenotype to another [3]. HIV-1 tropisms (R5, X4, R5X4) are often (but not always) defined in the following way. R5 viruses are those that can use only the CCR5 coreceptor and X4 viruses are those that can use only the CXCR4 coreceptor. R5X4 viruses, called dual-tropic viruses, can use both coreceptors. Tropism switch occurs during progression towards AIDS. Recently, it has been shown that R5 and X4 viruses modulate differentially host gene expression [4].

### Computer-aided prediction

The simplest method used for HIV-1 coreceptor usage prediction is known as the *charge rule *[5,6]. It relies only on the charge of residues at positions 11 and 25 within the V3 loop aligned against a consensus. The V3 loop is the third highly variable loop in the retroviral envelope protein gp120. Nonetheless, other positions are also important since the removal of these positions gave predictors with comparable (but weaker) performance to those that were trained with these positions present [1]. Other studies [7-12] also outlined the importance of other positions and proposed machine learning algorithms, such as the random forest [11] and the support vector machine (SVM) with structural descriptors [10], to built better predictors (than the charge rule). Available predictors (through webservers) of HIV-1 coreceptor usage are enumerated in [13].

An accuracy of 91.56% for the task of predicting the CXCR4 usage was obtained by [10]. Their method, based on structural descriptors of the V3 loop, employed a single dataset containing 432 sequences without indels and required the multiple alignment of all V3 sequences. However, such a prior alignment before learning might remove information present in the sequences which is relevant to the coreceptor usage task. Furthermore, a prior multiple alignment done on all the data invalidates the cross-validation method since the testing set in each fold has been used for the construction of the tested classifier. Another drawback of having an alignment-based method is that sequences having too many indels (when compared to a consensus sequence) are discarded to prevent the multiple alignment from yielding an unacceptable amount of gaps. In this paper, we present a method for predicting the coreceptor usage of HIV-1 which does not perform any multiple alignment prior to learning.

The SVM [14] has proven to be very effective at generating classifiers having good generalization (i.e., having high predicting accuracy). In particular, [1] have obtained a significantly improved predictor (in comparison with the charge rule) with an SVM equipped with a linear kernel. However, the linear kernel is not suited for sequence classification since it does not provide a natural measure of dissimilarity between sequences. Moreover, a SVM with a linear kernel can only use sequences that are exactly of the same length. Consequently, [1] aligned all HIV-1 V3 loop sequences with respect to a consensus. No such alignment was performed in our experiments. In contrast, string kernels [15] do not suffer from these deficiencies and have been explicitly designed to deal with strings and sequences of varying lengths. Furthermore, they have been successfully used for protein homology detection [16] – a classification problem which is closely related to the one treated in this paper.

Consequently, we have investigated the performance of the SVM, equipped with the appropriate string kernel, at predicting the coreceptor used by HIV-1 as a function of its protein envelope sequence (the V3 loop). We have compared two string kernels used for protein homology detection, namely the blended spectrum kernel [15,17] and the local alignment kernel [16], to a newly proposed string kernel, that we called the *distant segments *(DS) kernel.

### Applications

Bioinformatic methods for predicting HIV phenotypes have been tested in different situations and the concordance is high [18-21].

As described in [18], current bioinformatics programs are underestimating the use of CXCR4 by dual-tropic viruses in the brain. In [19], a concordance rate of 91% was obtained between genotypic and phenotypic assays in a clinical setting of 103 patients. In [20], the authors showed that the SVM with a linear kernel achieves a concordance of 86.5% with the Trofile assay and a concordance of 79.7% with the TRT assay. Recombinant assays (Trofile and TRT) are described in [20].

Further improvements in available HIV classifiers could presumably allow the replacement of in vitro phenotypic assays by a combination of sequencing and machine learning to determine the coreceptor usage. DNA sequencing is cheap, machine learning technologies are very accurate whereas phenotypic assays are labor-intensive and take weeks to produce readouts [13]. Thus, the next generation of bioinformatics programs for the prediction of coreceptor usage promises major improvements in clinical settings.

## Methods

We used the SVM to predict the coreceptor usage of HIV-1 as a function of its protein envelope sequence. The SVM is a discriminative learning algorithm used for binary classification problems. For these problems, we are given a *training set *of *examples*, where each example is labelled as being either *positive *or *negative*. In our case, each example is a string *s *of amino acids. When the binary classification task consists of predicting the usage of CCR5, the label of string *s *is +1 if *s *is the V3 loop of the protein envelope sequence of a HIV-1 virion that uses the CCR5 coreceptor, and -1 otherwise. The same method applies for the prediction of the CXCR4 coreceptor usage. When the binary classification task consists of predicting the capability of utilizing CCR5 and CXCR4 coreceptors, the label of string *s *is +1 if *s *is the V3 loop of the protein envelope sequence of a HIV-1 virion that uses both the CCR5 and CXCR4 coreceptors, and -1 if it is a virion that does not use CCR5 or does not use CXCR4.

Given a training set of binary labelled examples, each generated according to a fixed (but unknown) distribution *D*, the task of the learning algorithm is to produce a classifier *f *which will be as accurate as possible at predicting the correct class *y *of a test string *s *generated according to *D *(*i.e*., the same distribution that generated the training set). More precisely, if *f *(*s*) denotes the output of classifier *f *on input string *s*, then the task of the learner is to find *f *that minimizes the probability of error $\underset{(s,y)\sim D}{\mathrm{Pr}}(f(s)\neq y)$. A classifier *f *achieving a low probability of error is said to *generalize *well (on examples that are not in the training set).

To achieve its task, the learning algorithm (or learner) does not have access to the unknown distribution *D*, but only to a limited set of training examples, each generated according to *D*. It is still unknown exactly what is best for the learner to optimize on the training set, but the learning strategy used by the SVM currently provides the best empirical results for many practical binary classification tasks. Given a training set of labelled examples, the learning strategy used by the SVM consists at finding a soft-margin hyperplane [14,22], in a feature space of high dimensionality, that achieves the appropriate trade-off between the number of training errors and the magnitude of the separating margin realized on the training examples that are correctly classified (see, for example, [15]).

In our case, the SVM is used to classify strings of amino acids. The feature space, upon which the separating hyperplane is built, is defined by a mapping from each possible string *s *to a high-dimensional vector *ϕ *(*s*). For example, in the case of the *blended spectrum kernel *[15], each component *ϕ*_*α *_(*s*) is the frequency of occurrence in *s *of a specific substring *α *that we call a *segment*. The whole vector *ϕ *(*s*) is the collection of all these frequencies for each possible segment of at most *p *symbols. Consequently, vector *ϕ *(*s*) has $\sum_{i=1}^{p}|\Sigma {|}^{i}$ components for an alphabet Σ containing |Σ| symbols. If *w *denotes the normal vector of the separating hyperplane, and *b *its bias (which is related to the distance that the hyperplane has from the origin), then the output *f *(*s*) of the SVM classifier, on input string *s*, is given by

*f *(*s*) = sgn (⟨*w*, *ϕ *(*s*)⟩ + *b*),

where sgn(*a*) = +1 if *a *> 0 and -1 otherwise, and where ⟨*w*, *ϕ *(*s*)⟩ denotes the inner product between vectors *w *and *ϕ *(*s*). We have ⟨*w*, *ϕ *(*s*)⟩ = $\langle w,\phi (s)\rangle =\sum_{i=1}^{d}w_{i}\phi_{i}(s)$ for *d*-dimensional vectors. The normal vector *w *is often called the *discriminant *or the *weight vector*.

### Learning in spaces of large dimensionality

Constructing a separating hyperplane in spaces of very large dimensionality has potentially two serious drawbacks. The first drawback concerns the obvious danger of *overfitting*. Indeed, with so many degrees of freedom for a vector *w *having more components than the number of training examples, there may exist many different *w *having a high probability of error while making very few training errors. However, several theoretical results [15,22] indicate that overfitting is unlikely to occur when a large separating margin is found on the (numerous) correctly classified examples – thus giving theoretical support to the learning strategy used by the SVM.

The second potential drawback concerns the computational cost of using very high dimensional feature vectors *ϕ *(*s*_1_), *ϕ *(*s*_2_),..., *ϕ*(*s*_*m*_) of training examples. As we now demonstrate, this drawback can elegantly be avoided by using *kernels *instead of feature vectors. The basic idea consists of representing the discriminant *w *as a linear combination of the feature vectors of the training examples. More precisely, given a training set {(*s*_1_, *y*_1_), (*s*_2_, *y*_2_),..., (*s*_*m*_, *y*_*m*_)} and a mapping *ϕ *(·), we write $w=\sum_{i=1}^{m}\alpha_{i}y_{i}\phi (s_{i})$. The set {*α*_1_,..., *α*_*m*_} is called the *dual representation *of the (primal) weight vector *w*. Consequently, the inner product ⟨*w*, *ϕ *(*s*)⟩, used for computing the output of an SVM classifier, becomes

$$\langle w,\phi (s)\rangle =\sum_{i=1}^{m}\alpha_{i}y_{i}\langle \phi (s_{i}),\phi (s)\rangle =\sum_{i=1}^{m}\alpha_{i}y_{i}k(s_{i},s),$$

where $k(s,t)\overset{\text{def}}{\underset{}{=}}\langle \phi (s),\phi (t)\rangle$ defines the *kernel function *associated with the feature map *ϕ *(·). With the dual representation, the SVM classifier is entirely described in terms of the training examples *s*_*i *_having a non-zero value for *α*_*i*_. These examples are called *support vectors*. The so-called "kernel trick" consists of using *k *(*s*, *t*) without explicitly computing ⟨*ϕ *(*s*), *ϕ *(*t*)⟩ – a computationally prohibitive task for feature vectors of very large dimensionality. This is possible for many feature maps *ϕ *(·). Consider again, for example, the *blended spectrum *(BS) kernel where each component *ϕ*_*α *_(*s*) is the frequency of occurrence of a segment *α *in string *s *(for all words of at most *p *characters of an alphabet Σ). In this case, instead of performing $\sum_{i=1}^{p}|\Sigma {|}^{i}$ multiplications to compute explicitly ⟨*ϕ *(*s*), *ϕ *(*t*)⟩, we can compute, for each position *i *in string *s *and each position *j *in string *t*, the number of consecutive symbols that matches in *s *and *t*. We use the big-Oh notation to provide an upper bound to the running time of algorithms. Let *T *(*n*) denote the execution time of an algorithm on an input of size *n*. We say that *T *(*n*) is in *O *(*g *(*n*)) if and only if there exists a constant *c *and a critical *n*_0 _such that *T *(*n*) ≤ *cg *(*n*) for all *n *≥ *n*_0_. The blended spectrum kernel requires at most *O *(*p*·|*s*|·|*t*|) time for each string pair (*s*, *t*) – an enormous improvement over the Ω (|Σ|^*p*^) time required for the explicit computation of the inner product between a pair of feature vectors. In fact, there exists an algorithm [15] for computing the blended spectrum kernel in *O *(*p*·max (|*s*|, |*t*|)) time.

### The distant segments kernel

The blended spectrum kernel is interesting because it contains all the information concerning the population of segments that are present in a string of symbols without considering their relative positions. Here, we propose the *distant segments *(DS) kernel that, in some sense, extends the BS kernel to include (relative) positional information of segments in a string of symbols.

If one considers the frequencies of all possible segment distances inside a string as its features, then a precise comparison can be done between any pair of strings. Remote protein homology can be detected using distances between polypeptide segments [23]. For any string *s *of amino acids, these authors used explicitly a feature vector *ϕ *(*s*) where each component *ϕ*_*d*, *α*, *α' *_(*s*) denotes the number of times the (polypeptide) segment *α' *is located at distance *d *(in units of symbols) following the (polypeptide) segment *α*. They have restricted themselves to the case where *α *and *α' *have the same length *p*, with *p *≤ 3. Since the distance *d *is measured from the first symbol in *α *to the first symbol in *α'*, the *d *= 0 components of *ϕ *(*s*), *i.e*., *ϕ*_0,*α*,*α' *_(*s*), are non-zero only for *α *= *α' *and represent the number of occurrences of segment *α *in string *s*. Consequently, this feature vector strictly includes all the components of the feature vector associated with the BS kernel but is limited to segments of size *p *(for *p *≤ 3). By working with the explicit feature vectors, these authors were able to obtain easily the components of the discriminant vector *w *that are largest in magnitude and, consequently, are the most relevant for the binary classification task. However, the memory requirement of their algorithm increases exponentialy in *p*. Not surprisingly, only the results for *p *≤ 3 were reported by [23].

Despite these limitations, the results of [23] clearly show the relevance of having features representing the frequency of occurrences of pairs of segments that are separated by some distance for protein remote homology detection. Hence, we propose in this section the *distance segments *(DS) kernel that potentially includes all the features considered by [23] without limiting ourselves to *p *≤ 3 and to the case where the words (or segments) have to be of the same length. Indeed, we find no obvious biological motivation for these restrictions. Also, as we will show, there is no loss of interpretability of the results by using a kernel instead of the feature vectors. In particular, we can easily obtain the most significant components of the discriminant *w *by using a kernel. We will show that the time and space required for computing the kernel matrix and obtaining the most significant components of the discriminant *w *are bounded polynomially in terms of all the relevant parameters.

Consider a protein as a string of symbols from the alphabet Σ of amino acids. Σ* represents the set of all finite strings (including the empty string). For *μ *∈ Σ*, |*μ*| denotes the length of the string *μ*. Throughout the paper, *s*, *t*, *α*, *μ *and *ν *will denote strings of Σ*, whereas *θ *and *δ *will be lengths of such strings. Moreover, *μ ν *will denote the concatenation of *μ *and *ν*. The DS kernel is based on the following set. Given a string *s*, let $S_{\alpha ,\alpha^{\prime }}^{\delta }(s)$ be the set of all the occurrences of substrings of length *δ *that are beginning by segment *α *and ending by segment *α'*. More precisely,

(1) $$S_{\alpha ,\alpha^{\prime }}^{\delta }(s)\overset{\text{def}}{\underset{}{=}}\{(\mu ,\alpha ,\nu ,\alpha^{\prime },\mu^{\prime }):s=\mu \alpha \nu \alpha^{\prime }\mu^{\prime }\wedge 1\leq |\alpha |\wedge 1\leq |\alpha^{\prime }|\wedge 0\leq |\nu |\wedge \delta =|s|-|\mu |-|\mu^{\prime }|\}.$$

Note that the substring length *δ *is related to the distance *d *of [23] by *δ *= *d *+ |*α'*| where *d *= |*α*| + |*ν*| when |*α*| and |*α'*| do not overlap. Note also that, in contrast with [23], we may have |*α*| ≠ |*α'*|. Moreover, the segments *α *and *α' *never overlap since *μανα' μ' *equals to the whole string *s *and 0 ≤ |*ν*|. We have made this choice because it appeared biologically more plausible to have a distance ranging from the end of the first segment to the beginning of the second segment. Nevertheless, we will see shortly that we can include the possibility of overlap between segments with a very minor modification of the kernel.

The DS kernel is defined by the following inner product

(2) $$k_{DS}^{\delta_{m},\theta_{m}}(s,t)\overset{\text{def}}{\underset{}{=}}\langle \phi_{DS}^{\delta_{m},\theta_{m}}(s),\phi_{DS}^{\delta_{m},\theta_{m}}(t)\rangle ,$$

where $\phi_{DS}^{\delta_{m},\theta_{m}}(s)$ is the feature vector

$$\phi_{DS}^{\delta_{m},\theta_{m}}(s)\overset{\text{def}}{\underset{}{=}}{\left(\left|S_{\alpha ,\alpha^{\prime }}^{\delta }(s)\right|\right)}_{\left\{(\delta ,\alpha ,\alpha^{\prime }):1\leq |\alpha |\leq \theta_{m}\wedge 1\leq |\alpha^{\prime }|\leq \theta_{m}\wedge |\alpha |+|\alpha^{\prime }|\leq \delta \leq \delta_{m}\right\}}.$$

**Figure 1:**
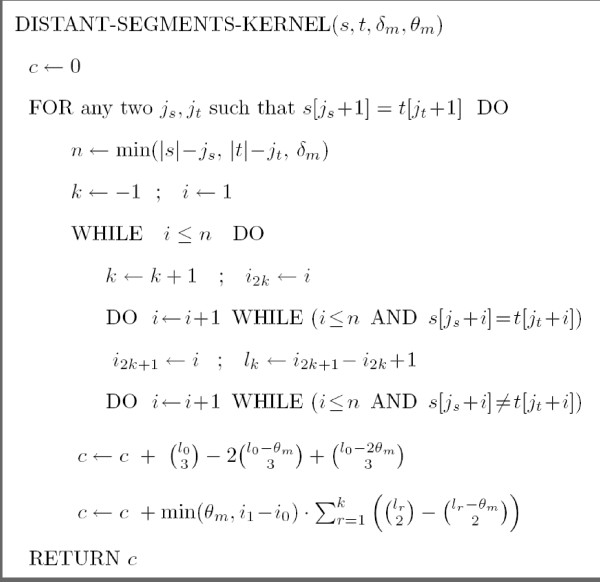
The algorithm for computing $k_{DS}^{\delta_{m},\theta_{m}}(s,t)$.

Hence, the kernel is computed for a fixed maximum value *θ*_*m *_of segment sizes and a fixed maximum value *δ*_*m *_of substring length. Note that, the number of strings of size *θ *of Σ* grows exponentially with respect to *θ*. Fortunately, we are able to avoid this potentially devastating combinatorial explosion in our computation of $k_{DS}^{\delta_{m},\theta_{m}}(s,t)$. Figure 1 shows the pseudo-code of the algorithm. In the pseudo-code, *s *[*i*] denotes the symbol located at position *i *in the string *s *(with *i *∈ {1, 2,..., |*s*|}). Moreover, for any integers *i*, *j*, $\left(\begin{array}{c} j\\ i \end{array}\right)$ denotes $\frac{j!}{i!(j-i)!}$ if 0 ≤ *i *≤ *j*, and 0 otherwise. Admittedly, it is certainly not clear that the algorithm of Figure 1 actually computes the value of $k_{DS}^{\delta_{m},\theta_{m}}(s,t)$ given by Equation 2. Hence, a proof of correctness of this algorithm is presented at the appendix (located after the conclusion). The worst-case running time is easy to obtain because the algorithm is essentially composed of three imbricated loops: one for *j*_*s *_∈ {0,..., |*s*|-1}, one for *j*_*t *_∈ {0,..., |*t*|-1}, and one for *i *∈ {1,..., min(|*s*|, |*t*|, *δ*_*m*_)}. The time complexity is therefore in *O *(|*s*|·|*t*|·min(|*s*|, |*t*|, *δ*_*m*_)).

Note that the definition of the DS-kernel can be easily modified in order to accept overlaps between *α *and *α'*. Indeed, when overlaps are permitted, they can only occur when both *α *and *α' *start and end in {*j*_*s *_+ *i*_0_,..., *j*_*s *_+ *i*_1_-1}. The number of elements of $S_{\left(j_{s},j_{t}\right)}$ for which *i*_2*r *_≤ *δ *<*i*_2*r*+1 _is thus the same for all values of *r*, including *r *= 0. Consequently, the algorithm to compute the DS kernel, when overlaps are permitted, is the same as the one in Figure 1 except that we need the replace the last two lines of the FOR loop, involved in the computation of *c*, by the single line:

$$c\leftarrow c+\min(\theta_{m},i_{1}-i_{0})\cdot \sum_{r=0}^{k}\left(\left(\begin{array}{c} l_{r}\\ 2 \end{array}\right)-\left(\begin{array}{c} l_{r}-\theta_{m}\\ 2 \end{array}\right)\right).$$

Similar simple modifications can be performed for the more restrictive case of |*α*| = |*α'*|.

### Extracting the discriminant vector with the distant segments kernel

**Figure 2:**
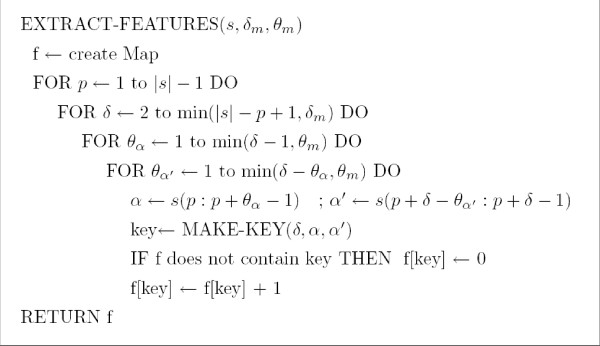
The algorithm for extracting the features of a string *s *into a Map. Here, *s *(*i *: *j*) denotes the substring of *s *starting at position *i *and ending at position *j*.

**Figure 3:**
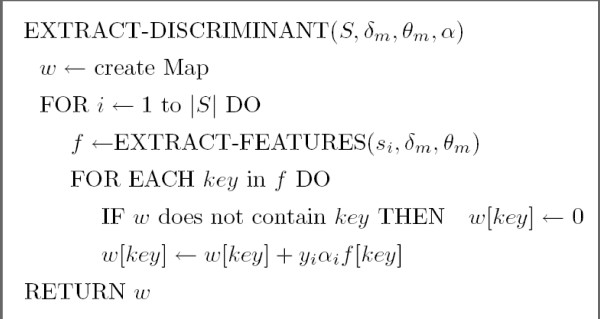
The algorithm for merging every feature from the set *S *= {(*s*_1_, *y*_1_), (*s*_2_, *y*_2_), ..., (*s*_*l*_, *y*_*l*_)} of all support vectors into a Map representing the discriminant *w*.

We now show how to extract (with reasonable time and space resources) the components of the discriminant *w *that are non-zero. Recall that $w=\sum_{i=1}^{l}\alpha_{i}y_{i}\phi (s_{i})$ when the SVM contains *l *support vectors {(*s*_1_, *y*_1_),..., (*s*_*l*_, *y*_*l*_)}. Recall also that each feature *ϕ*_*δ*, *α*, *α' *_(*s*_*i*_) is identified by a triplet (*δ*, *α*, *α'*), with *δ *≥ |*α*| + |*α'*|. Hence, to obtain the non-zero valued components of *w*, we first obtain the non-zero valued features *ϕ*_*δ*, *α*, *α' *_(*s*_*i*_) from each support vector (with Algorithm EXTRACT-FEATURES of Figure 2) and then collect and merge every feature of each support vector by multiplying each of them by *α*_*i*_*y*_*i *_(with Algorithm EXTRACT-DISCRIMINANT of Figure 3).

We transform each support vector *ϕ *(*s*_*i*_) into a *Map *of features. Each *Map key *is an identifier for a (*δ*, *α*, *α'*) having *ϕ*_*δ*, *α*, *α' *_(*s*_*i*_) > 0. The *Map value *is given by *ϕ*_*δ*, *α*, *α'*_(*s*_*i*_) for each key.

The worst-case access time for an AVL-tree-Map of *n *elements is *O *(log *n*). Hence, from Figure 2, the time complexity of extracting all the (non-zero valued) features of a support vector is in $O(|s|\theta_{m}^{2}\delta_{m}\cdot \log(|s|\theta_{m}^{2}\delta_{m}))$. Moreover, since the total number of features inserted to the Map by the algorithm EXTRACT-DISCRIMINANT is at most $l\cdot |s|\cdot \theta_{m}^{2}\delta_{m}$, the time complexity of extracting all the non-zero valued components of *w *is in $O(l|s|\theta_{m}^{2}\delta_{m}\cdot \log(l|s|\theta_{m}^{2}\delta_{m}))$.

### SVM

We have used a publicly available SVM software, named SVM^light ^[24], for predicting the coreceptor usage. Learning SVM classifier requires to choose the right trade-off between training accuracy and the magnitude of the separating margin on the correctly classified examples. This trade-off is generally captured by a so-called soft-margin hyperparameter *C*.

The learner must choose the value of *C *from the training set only – the testing set must be used only for estimating the performance of the final classifier. We have used the (well-known) 10-fold cross-validation method (on the training set) to determine the best value of *C *and the best values of the kernel hyperparameters (that we describe below). Once the values of all the hyperparameters were found, we used these values to train the final SVM classifier on the whole training set.

### Selected metrics

The testing of the final SVM classifier was done according to several metrics. Let P and N denote, respectively, the number of positive examples and the number of negative examples in the test set. Let TP, the number of "true positives", denote the number of positive testing examples that are classified (by the SVM) as positive. A similar definition applies to TN, the number of "true negatives". Let FP, the number of "false positives", denote the number of negative testing examples that are classified as positive. A similar definition applied to FN, the number of "false negatives". To quantify the "fitness" of the final SVM classifier, we have computed the *accuracy*, which is (TP+TN)/(P+T), the *sensitivity*, which is TP/P, and the specificity, which is TN/N. Finally, for those who cannot decide how much to weight the cost of a false positive, in comparison with a false negative, we have computed the "area under the ROC curve" as described by [25].

Unlike the other metrics, the accuracy (which is 1 – the testing error) has the advantage of having very tight confidence intervals that can be computed straightforwardly from the binomial tail inversion, as described by [26]. We have used this method to find if whether or not the observed difference of testing accuracy (between two classifiers) was statistically significant. We have reported the results only when a statistically significant difference was observed with a 90% confidence level.

### Selected string kernels

One of the kernel used was the blended spectrum (BS) kernel that we have described above. Recall that the feature space, for this kernel, is the count of all *k*-mers with 1 ≤ *k *≤ *p*. Hence *p *is the sole hyperparameter of this kernel.

We have also used the local alignment (LA) kernel [16] which can be thought of as a soft-max version of the Smith-Waterman local alignment algorithm for pair of sequences. Indeed, instead of considering the alignment that maximizes the Smith-Waterman (SW) score, the LA kernel considers every local alignment with a Gibbs distribution that depends on its SW score. Unfortunately, the LA kernel has too many hyperparameters precluding their optimization by cross-validation. Hence, a number of choices were made based on the results of [16]. Namely, the alignment parameters were set to (BLOSUM 62, *e *= 11, *d *= 1) and the empirical kernel map of the LA kernel was used. The hyperparameter *β *was the only one that was adjusted by cross-validation.

Of course, the proposed distant segments (DS) kernel was also tested. The *θ*_*m *_hyperparameter was set to *δ*_*m *_to avoid the limitation of segment length. Hence, *δ*_*m *_was the sole hyperparameter for this kernel that was optimized by cross-validation.

Other interesting kernels, not considered here because they yielded inferior results (according to [16], and [23]) on the remote protein homology detection problem, include the mismatch kernel [27] and the pairwise kernel [28].

### Datasets

The V3 loop sequence and coreceptor usage of HIV-1 samples were retrieved from Los Alamos National Laboratory HIV Databases  through available online forms.

Every sample had a unique GENBANK identifier. Sequences containing #, $ or * were eliminated from the dataset. The signification of these symbols was reported by Brian Foley of Los Alamos National Laboratory (personal communication). The # character indicates that the codon could not be translated, either because it had a gap character in it (a frame-shifting deletion in the virus RNA), or an ambiguity code (such as R for purine). The $ and * symbols represent a stop codon in the RNA sequence. TAA, TGA or TAG are stop codons. The dataset was first shuffled and then splitted half-half, yielding a training and a testing set. The decision to shuffle the dataset was made to increase the probability that both the training and testing examples are obtained from the same distribution. The decision to use half of the dataset for testing was made in order to obtain tight confidence intervals for accuracy.

Samples having the same V3 loop sequence and a different coreceptor usage label are called *contradictions*. Contradictions were kept in the datasets to have prediction performances that take into account the biological reality of dual tropism for which frontiers are not well defined.

Statistics were compiled for the coreceptor usage distribution, the count of contradictions, the amount of samples in each clades and the distribution of the V3 loop length.

## Results

Here we report statistics on our datasets, namely the distribution, contradictions, subtypes and the varying lengths. We also show the results of our classifiers on the HIV-1 coreceptor usage prediction task, a brief summary of existing methods and an analysis of the discriminant vector with the distant segments kernel.

### Statistics

In Table 1 is reported the distribution of coreceptor usages in the datasets created from Los Alamos National Laboratory HIV Databases data. In the training set, there are 1225 CCR5-utilizing samples (85.9%), 375 CXCR4-utilizing samples (26.3%) and 175 CCR5-and-CXCR4-utilizing samples (12.2%). The distribution is approximatly the same in the test set. There are contradictions (entries with the same V3 sequence and a different coreceptor usage) in all classes of our datasets. A majority of viruses can use CCR5 in our datasets.

**Table 1:** Datasets. Contradictions are in parenthesis.

Coreceptor usage	Training set			Test set		
	Negative examples	Positive examples	Total	Negative examples	Positive examples	Total
CCR5	200 (13)	1225 (12)	1425 (25)	225 (22)	1200 (16)	1425 (38)
CXCR4	1050 (44)	375 (18)	1425 (62)	1027 (28)	398 (21)	1425 (38)
CCR5 and CXCR4	1250 (57)	175 (30)	1425 (87)	1252 (48)	173 (35)	1425 (83)

In Table 2, the count is reported regarding HIV-1 subtypes, also known as genetic clades. HIV-1 subtype B is the most numerous in our datasets. The clade information is not an attribute that we provided to our classifiers, we only built our method on the primary structure of the V3 loop. Therefore, our method is independant of the clades.

**Table 2:** HIV-1 subtypes.

Subtype	Training set	Test set	Total
A	39	46	85
B	955	943	1898
C	168	149	317
02_AG	12	15	27
O	11	11	22
D	69	95	164
A1	25	18	43
AG	5	5	10
01_AE	97	106	203
G	7	7	14
Others	37	30	67

Total	1425	1425	2850

The V3 loops have variable lengths among the virions of a population. In our dataset (Table 3), the majority of sequences has exactly 36 residues, although the length ranges from 31 to 40.

**Table 3:** Sequence length distribution. The minimum length is 31 residues and the maximum length is 40 residues.

Residues	Training set	Test set	Total
31	1	0	1
32	0	0	0
33	2	2	4
34	18	22	40
35	210	189	399
36	1142	1162	2304
37	30	31	61
38	11	10	21
39	11	8	19
40	0	1	1

Total	1425	1425	2850

### Coreceptor usage predictions

Classification results on the three different tasks (CCR5, CXCR4, CCR5-and-CXCR4) are presented in Table 4 for three different kernels.

**Table 4:** Classification results on the test sets. Accuracy, specificity and sensitivity are defined in Methods. See [25] for a description of the ROC area.

Coreceptor usage	SVM parameter C	Kernel parameter	Support vectors	Accuracy	Specificity	Sensitivity	ROC area
**Blended spectrum kernel**							
CCR5	0.04	3	204	96.63%	85.33%	98.75%	98.68%
CXCR4	0.7	9	392	93.68%	96.00%	87.68%	96.59%
CCR5 and CXCR4	2	15	430	94.38%	98.16%	67.05%	90.16%

**Local alignment kernel**							
CCR5	9	1	200	96.42%	87.55%	98.08%	98.12%
CXCR4	0.02	0.05	321	92.21%	97.56%	78.39%	95.11%
CCR5 and CXCR4	0.5	0.1	399	92.28%	97.20%	56.64%	87.49%

**Distant segments kernel**							
CCR5	0.4	30	533	96.35%	83.55%	98.75%	98.95%
CXCR4	0.0001	30	577	94.80%	97.56%	87.68%	96.25%
CCR5 and CXCR4	0.2	35	698	95.15%	99.20%	65.89%	90.97%

**Perfect deterministic classifier**							
CCR5	-	-	-	99.15%	99.55%	99.08%	-
CXCR4	-	-	-	98.66%	99.70%	95.97%	-
CCR5 and CXCR4	-	-	-	97.96%	99.68%	85.54%	-

**Distant segments kernel trained on test set**							
CCR5	0.3	40	425	98.45%	92.88%	99.5%	99.17%
CXCR4	0.0001	35	611	98.66%	99.70%	95.97%	98.29%
CCR5 and CXCR4	0.0001	40	618	97.96%	99.68%	85.54%	96.27%

For the CCR5-usage prediction task, the SVM classifier achieved a testing accuracy of 96.63%, 96.42%, and 96.35%, respectively, for the BS, LA, and DS kernels. By using the binomial tail inversion method of [26], we find no statistically significant difference, with 90% confidence, between kernels.

For the CXCR4-usage prediction task, the SVM classifier achieved a testing accuracy of 93.68%, 92.21%, and 94.80%, respectively, for the BS, LA, and DS kernels. By using the binomial tail inversion method of [26], we find that the difference is statistically significant, with 90% confidence, for the DS versus the LA kernel.

For the CCR5-and-CXCR4-usage task, the SVM classifier achieved a testing accuracy of 94.38%, 92.28 %, and 95.15%, respectively, for the BS, LA, and DS kernels. Again, we find that the difference is statistically significant, with 90% confidence, for the DS versus the LA kernel.

Overall, all the tested string kernels perform well on the CCR5 task, but the DS kernel is significantly better than the LA kernel (with 90% confidence) for the CXCR4 and CCR5-and-CXCR4 tasks. For these two prediction tasks, the performance of the BS kernel was closer to the one obtained for the DS kernel than the one obtained for the LA kernel.

### Classification with the perfect deterministic classifier

Also present in Table 4 are the results of the *perfect deterministic classifier*. This classifier is the deterministic classifier achieving the highest possible accuracy on the test set. For any input string *s *in a testing set *T*, the perfect determinist classifier (h*) returns the most frequently encountered class label for string *s *in *T*. Hence, the accuracy on *T *of h* is an overall measure of the amount of contradictions that are present in *T*. There are no contradictions in *T *if and only if the testing accuracy of h* is 100%. As shown in Table 4, there is a significant amount of contradictions in the test set *T*. These results indicate that any deterministic classifier cannot achieve an accuracy greater than 99.15%, 98.66% and 97.96%, respectively for the CCR5, CXCR4, and CCR5-and-CXCR4 coreceptor usage tasks.

### Discriminative power

To determine if a SVM classifier equipped with the distant segments (DS) kernel had enough discriminative power to achieve the accuracy of perfect determinist classifier, we trained the SVM, equipped with the DS kernel, *on the testing set*. From the results of Table 4, we conclude that the SVM equipped with the DS kernel possess sufficient discriminative power since it achieved (almost) the same accuracy as the perfect deterministic classifier for all three tasks. Hence, the fact that the SVM with the DS kernel does not achieve the same accuracy as the perfect determinist classifier when it is obtained from the training set (as indicated in Table 4) is not due to a lack of discriminative power from the part of the learner.

### Discriminant vectors

**Figure 4:**
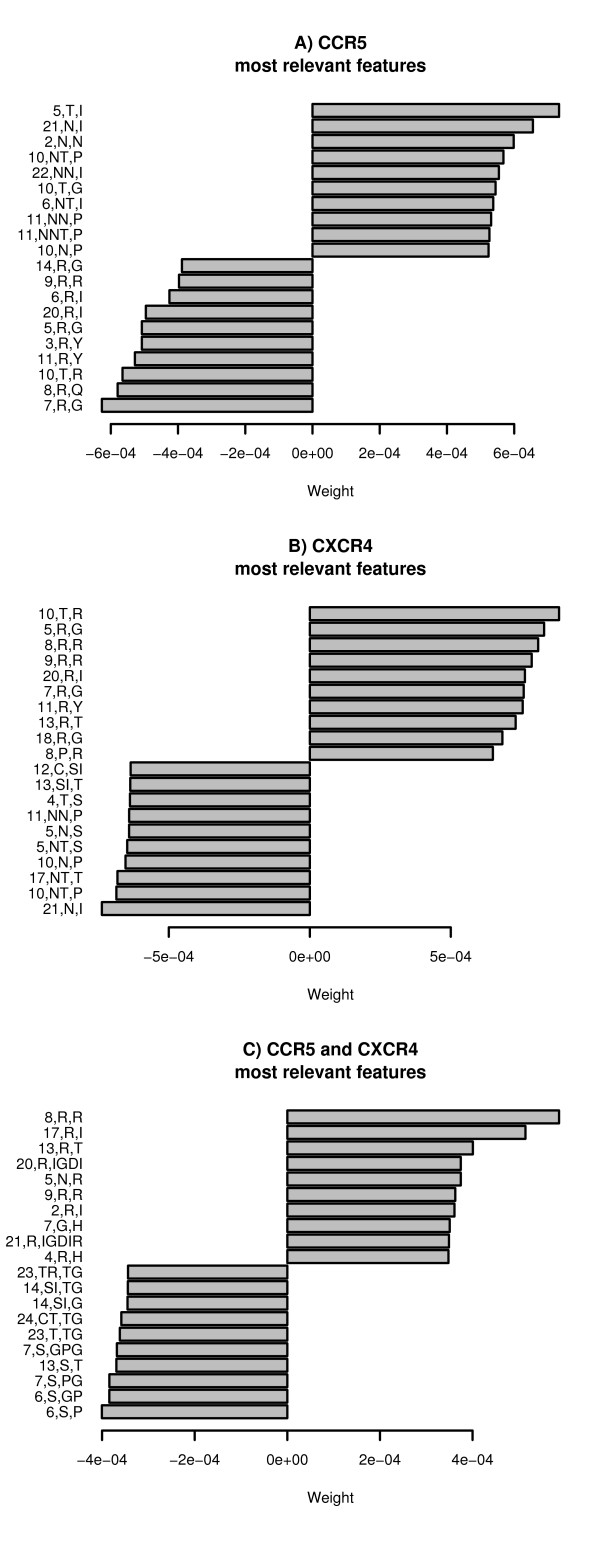
Features (20 are shown) with highest and lowest weights for each coreceptor usage prediction task.

The discriminant vector that maximizes the soft-margin has (almost always) many non-zero valued components which can be extracted by the algorithm of Figure 3. We examine which components of the discriminant vector have the largest absolute magnitude. These components give weight to the most relevant features for a given classification task. In Figure 4, we describe the most relevant features for each tasks. Only the 20 most significant features are shown.

A subset of positive-weighted features shown for CCR5-utilizing viruses are also in the negative-weighted features shown for CXCR4-utilizing viruses. Furthermore, a subset of positive-weighted features shown for CXCR4-utilizing viruses are also in the negative-weighted features reported for CCR5-utilizing viruses. Thus, CCR5 and CXCR4 discriminant models are complementary. However, since 3 tropisms exist (R5, X4 and R5X4), features contributing to CCR5-and-CXCR4 should also include some of the features contributing to CCR5 and some of the features contributing to CXCR4. Among shown positive-weighted features for CCR5-and-CXCR4, there are features that also contribute to CXCR4 ([8, R, R], [13, R, T], [9, R, R]). On another hand, this is not the case for CCR5. However, only the twenty most relevant features have been shown and there are many more features, with similar weights, that contribute to the discriminant vector. In fact, the classifiers that we have obtained depend on a very large number of features (instead of a very small subset of relevant features).

## Discussion

The proposed HIV-1 coreceptor-usage prediction tool achieved very high accuracy in comparison with other existing prediction methods. In view of the results of Pillai et al, we have shown that the SVM classification accuracy can be greatly improved with the usage of a string kernel. Surprisingly, the local alignment (LA) kernel, which makes an explicit use of biologically-motivated scoring matrices (such as BLOSUM 62), turns out to be outperformed by the blended spectrum (BS) and the distant segments (DS) kernels which do not try to exploit any concept of similarity between residues but rely, instead, on a very large set of easily-interpretable features. Thus, a weighted-majority vote over a very high number of simple features constitutes a very productive approach, that is both sensitive and specific to what it is trained for, and applies well in the field of viral phenotype prediction.

### Comparison with available bioinformatic methods

In Table 5, we show a summary of the available methods. The simplest method (the charge rule) has an accuracy of 87.45%. Thus, the charge rule is the worst method presented in table 5. The SVM with string kernels is the only approach without multiple alignments. Therefore, V3 sequences with many indels can be used with our method, but not with the other. These other methods were not directly tested here with our datasets because they all rely on multiple alignments. The purpose of those alignments is to produce a consensus and to yield transformed sequences having all the same length. As indicated by the size of the training set in those methods, sequences having larger indels were discarded, thus making these datasets smaller. Most of the methods rely on cross-validation to perform quality assessment but, as we have mentioned, this is problematic when multiple alignments are performed prior to learning, since, in these cases, the testing set in each fold is used for the construction of the tested classifier. It is also important to mention that the various methods presented in Table 5 do not produce predictors for the same coreceptor usage task. Indeed, the definition of X4 viruses is not always the same: some authors refer to it as CXCR4-only while other use it as CXCR4-utilizing. It is thus unfeasible to assess the fitness of these approaches, which are twisted by cross-validation, multiple alignments and heterogeneous dataset composition.

**Table 5:** Available methods. The results column contains the metric and what the classifier is predicting.

Reference	Learning method	Training set	Testing set	Multiple alignments	Results
Pillai et al. 2003	Charge rule [5,6]	271	-	yes	Accuracy (CXCR4): 87.45%
Resch et al. 2001	Neural networks	181	-	yes	Specificity (X4): 90.00%
Pillai et al. 2003	SVM	271	-	yes	Accuracy (CXCR4): 90.86%
Jensen et al. 2003	PSSM^1^	213	175	yes	Specificity (CXCR4): 96.00%
Jensen et al. 2006	PSSM	279	-	yes	Specificity (CXCR4): 94.00%^2^
Sander et al. 2007	SVM	432	-	yes	Accuracy (CXCR4): 91.56%
Xu et al. 2007	Random forests	651	-	yes	Accuracy (R5): 95.10%
Lamers et al. 2008	Neural networks	149	-	yes	Accuracy (R5X4): 75.50%
This manuscript	SVM	1425	1425	**no**	Accuracy (CXCR4): **94.80%**

^1^Position-specific scoring matrices

^2^Subtype C

The work by Lamers and colleagues [12] is the first development in HIV-1 coreceptor usage prediction regarding dual-tropic viruses. Using evolved neural networks, an accuracy of 75.50% was achieved on a training set of 149 sequences with the cross-validation method. However, the SVM equipped with the distant segments kernel reached an accuracy of 95.15% on a large test set (1425 sequences) in our experiments. Thus, our SVM outperforms the neural network described by Lamers and colleagues [12] for the prediction of dual-tropic viruses.

### Los Alamos National Laboratory HIV Databases

Although we used only the Los Alamos National Laboratory HIV Databases as our source of sequence information, it is notable that this data provider represents a meta-resource, fetching bioinformation from databases around the planet, namely GenBank (USA, ), EMBL (Europe, ) and DDBJ (Japan, ). Researchers cannot directly send their HIV sequences to LANL, but it is clear that this approach makes this database less likely to contain errors.

### Noise

The primary cause of contradictions (e.g. a sequence having two or more phenotypes) remains uncharacterized. It may be due to a particular mix, to some extent, of virion envelope attributes (regions other than the V3) and of the host cell receptor counterparts. As genotypic assays, based on bioinformatics prediction software, rely on sequencing technologies, they are likely to play a more important role in clinical settings as sequencing cost drops. Next-generation sequencing platforms promise a radical improvement on the throughput and more affordable prices. Meanwhile, effective algorithmic methods with proven statistical significance must be developed. Bioinformatics practitioners have to innovate by creating new algorithms to deal with large datasets and need to take into consideration sequencing errors and noise in phenotypic assay readouts. Consequently, we investigated the use of statistical machine learning algorithms, such as the SVM, whose robustness against noise has been observed in many classification tasks. The high accuracy results we have obtained here indicate that this is also the case for the task of predicting the coreceptor usage of HIV-1. While it remains uncertain whether or not other components of the HIV-1 envelope contribute to the predictability of the viral phenotype, we have shown that the V3 loop alone produces very acceptable outputs despite the presence of a small amount of noise.

### Web server

To allow HIV researchers to test our method on the web, we have implemented a web server for the HIV-1 coreceptor usage prediction. The address of this web server is . In this setting, one has to submit fasta-formatted V3 sequences in a web form. Then, using the dual representation of the SVM with the distant segments kernel, the software predicts the coreceptor usage of each submitted viral sequence. Those predictions are characterized by high accuracy (according to results in Table 4). Source codes for the web server and for a command-line back-end are available in additional file 1.

## Conclusion

To our knowledge, this is the first paper that investigates the use of string kernels for predicting the coreceptor usage of HIV-1. Our contributions include a novel string kernel (the distant segments kernel), a SVM predictor for HIV-1 coreceptor usage with the identification of the most relevant features and state-of-the-art results on accuracy, specificity, sensitivity and receiver operating characteristic. As suggested, string kernels outperform all published algorithms for HIV-1 coreceptor usage prediction. Large margin classifiers and string kernels promise improvements in drug selection, namely CCR5 coreceptor inhibitors and CXCR4 coreceptor inhibitors, in clinical settings. Since the binding of an envelope protein to a receptor/coreceptor prior to infection is not specific to HIV-1, one could extend this work to other diseases. Furthermore, most ligand interactions could be analyzed in such a fashion. Detailed features in primary structures (DNA or protein sequences) can be elucidated with the proposed bioinformatic method. Although we have exposed that even the perfect algorithm (entitled "Perfect determinist classifier") can not reach faultless outcomes, we have also empirically demonstrated that our algorithms are very competitive (more than 96% with distant segments for CCR5). It is thus feasible to apply kernel methods based on features in primary structures to compare sequence information in the perspective of predicting a phenotype. The distant segments kernel has broad applicability in HIV research such as drug resistance, coreceptor usage (as shown in this paper), immune escape, and other viral phenotypes.

## Appendix

### Proof of the correctness of the distant segments kernel

We now prove that the algorithm DISTANT-SEGMENTS-KERNEL (*s*, *t*, *δ*_*m*_, *θ *_*m*_) does, indeed, compute $k_{DS}^{\delta_{m},\theta_{m}}(s,t)$ as defined by Equation 2.

*Proof*. For each pair (*j*_*s*_, *j*_*t*_) such that *s *[*j*_*s *_+ 1] = *t *[*j*_*t *_+ 1] and each *δ *≥ 2, let us define $S_{\left(j_{s},j_{t}\right)}$ to be the set of all triples (*δ*, (*μ*_*s*_, *α*, *ν*_*s*_, *α'*, ${\mu^{\prime }}_{s}$), (*μ*_*t*_, *α*, *ν*_*t*_, *α'*, ${\mu^{\prime }}_{t}$)) such that

$$\begin{array}{ll} -\delta \leq \delta_{m}; & \\ -|\alpha |\leq \theta_{m}\text{ and }|\alpha^{\prime }|\leq \theta_{m}; & \\ -(\mu_{u},\alpha ,\nu_{u},\alpha^{\prime },{\mu^{\prime }}_{u})\in S_{\alpha ,\alpha^{\prime }}^{\delta }(u) & \text{for }u=s\text{ and for }u=t;\\ -|\mu_{s}|=j_{s}\text{ and }|\mu_{t}|=j_{t}. & \end{array}$$

Clearly, $k_{DS}^{\delta_{m},\theta_{m}}(s,t)$ is the sum of all the values of |$S_{\left(j_{s},j_{t}\right)}$| over all the possible pairs (*j*_*s*_, *j*_*t*_). Moreover, it is easy to see that |$S_{\left(j_{s},j_{t}\right)}$| can be computed only from the knowledge of the set of indices *i *∈ {1,..., *n*} satisfying property *P *(*i*) : = *s *[*j*_*s *_+ *i*] = *t *[*j*_*t *_+ *i*]. Note that when the test of the first WHILE loop is performed, the value of *i *is such that *P *(*i*) is valid but not *P *(*i *- 1). Moreover, in the second WHILE loop, *P *(*i*) remains valid, except for the last test. Thus, *s *[*j*_*s *_+ *i*] = *t *[*j*_*t *_+ *i*] if and only if *i*_2*r *_≤ *i *<*i*_2*r*+1 _for some *r *∈ {0,..., *k*}. This, in turn, implies that each element of $S_{\left(j_{s},j_{t}\right)}$ must be such that *i*_2*r *_≤ *δ *<*i*_2*r*+1 _for some *r*. To obtain the result, it is therefore sufficient to prove that both of theses properties hold.

**P1 **The number of elements of $S_{\left(j_{s},j_{t}\right)}$, for which *i*_0 _<*δ *<*i*_1_, is given by

$$\left(\begin{array}{c} l_{0}\\ 3 \end{array}\right)-2\left(\begin{array}{c} l_{0}-\theta_{m}\\ 3 \end{array}\right)+\left(\begin{array}{c} l_{0}-2\theta_{m}\\ 3 \end{array}\right).$$

**P2 **For *r *∈ {1,..., *k*}, the number of elements of $S_{\left(j_{s},j_{t}\right)}$, for which *i*_2*r *_≤ *δ *<*i*_2*r*+1_, is given by

$$\min(\theta_{m},i_{1}-i_{0})\cdot \left(\left(\begin{array}{c} l_{r}\\ 2 \end{array}\right)-\left(\begin{array}{c} l_{0}-\theta_{m}\\ 2 \end{array}\right)\right).$$

To prove these properties, we will use the fact that, if 1 ≤ *i *≤ *k*, then $\left(\begin{array}{c} k\\ i \end{array}\right)$ counts the number of sequences ⟨*a*_1_,..., *a*_*i*_⟩ satisfying 1 ≤ *a*_1 _<*a*_2 _< ⋯ <*a*_*i *_≤ *k *where {*a*_1_, *a*_2_, ..., *a*_*i*_} are *i *string positions. Moreover, for any substring *u *of *s*, let us denote by *b*_*u *_the starting position of *u *in *s*, and by *e*_*u *_the first position of *s *after the substring *u *(if *s *ends with *u*, choose *e*_*u *_= |*s*| + 1).

We first prove **P2**. Fix an *r*. Since *i*_0 _= 1, we have that any *s*-substring *α *of length ≤ *θ*_*m *_with *b*_*α *_= *j*_*s *_+ *i*_0 _and *e*_*α *_≤ *j*_*s *_+ *i*_1 _together with any *s*-substring *α' *of length ≤ *θ*_*m *_such that

*j*_*s *_+ *i*_2*r *_≤ *b*_*α' *_<*e*_*α' *_≤ *j*_*s *_+ *i*_2*r*+1_,

will give rise to exactly one element of $S_{\left(j_{s},j_{t}\right)}$ with *i*_2*r *_≤ *δ *<*i*_2*r*+1_. Conversely, each element $S_{\left(j_{s},j_{t}\right)}$ such that *i*_2*r *_≤ *δ *<*i*_2*r*+1 _will have an *α *and an *α' *with these properties. Since *α *has to start at *j*_*s *_+ *i*_0_, it is easy to see that the number of such possible *α *is exactly min(*θ*_*m*_, *i*_1 _- *i*_0_). Thus let us show that the number of possible *α' *is exactly $\left(\begin{array}{c} l_{r}\\ 2 \end{array}\right)-\left(\begin{array}{c} l_{r}-\theta_{m}\\ 2 \end{array}\right)$.

Since *l*_*r *_gives the number of positions from *j*_*s *_+ *i*_2*r *_to *j*_*s *_+ *i*_2*r*+1 _inclusively, $\left(\begin{array}{c} l_{r}\\ 2 \end{array}\right)$ counts all the possible, choices of *b*_*α' *_and *e*_*α' *_with *j*_*s *_+ *i*_2*r *_≤ *b*_*α' *_<*e*_*α' *_≤ *j*_*s *_+ *i*_2*r*+1_. Thus $\left(\begin{array}{c} l_{r}\\ 2 \end{array}\right)$ counts the number of possible strings *α' *of all possible lengths (including lengths > *θ*_*m*_). On another hand, the number of *α' *having a length > *θ*_*m*_. is equal to $\left(\begin{array}{c} l_{r}-\theta_{m}\\ 2 \end{array}\right)$. Indeed, if *l*_*r *_- *θ*_*m *_< 2, $\left(\begin{array}{c} l_{r}-\theta_{m}\\ 2 \end{array}\right)$ = 0 as wanted, and otherwise, there is a one-to-one correspondence between the set of all sequences ⟨*a*_1_, *a*_2_⟩ such that 1 ≤ *a*_1 _<*a*_2 _≤ *l*_*r *_- *θ*_*m *_and the set of all *α' *of length > *θ*_*m*_. The correspondence is obtained by putting *b*_*α' *_= *i*_2*r *_+ *a*_1 _- 1 and *e*_*α' *_= *i*_2*r *_+ *θ*_*m *_+ *a*_2 _- 1.

The proof for **P1 **is similar to the one for **P2 **except that we have to consider the fact that both *α *and *α' *start and end in {*j*_*s *_+ *i*_0_,..., *j*_*s *_+ *i*_1 _- 1}. Since no overlap is allowed and *b*_*α *_= *j*_*s *_+ *i*_0_, we must have

*j*_*s *_+ *i*_0 _≤ *e*_*α *_- 1 <*b*_*α' *_<*e*_*α' *_≤ *j*_*s *_+ *i*_1_.

Since *l*_0 _gives the number of positions from *j*_*s *_+ *i*_0 _to *j*_*s *_+ *i*_1 _inclusively, $\left(\begin{array}{c} l_{0}\\ 3 \end{array}\right)$ counts all the possible choices of *α *and *α' *for all possible lengths. Recall that if *l*_0 _< 3, which can only occur if *i*_1 _= *i*_0 _+ 1, we have that $\left(\begin{array}{c} l_{0}\\ 3 \end{array}\right)$, as wanted.

On another hand, $\left(\begin{array}{c} l_{0}-\theta_{m}\\ 3 \end{array}\right)$ counts all the possible choices of *α *of length > *θ*_*m *_and of *α' *of arbitrary length. This set of possible choices is non empty only if *l*_0 _- *θ*_*m *_≥ 3 and, then, the one-to-one correspondence between a sequence ⟨*a*_1_, *a*_2_, *a*_3_⟩ such that 1 ≤ *a*_1 _<*a*_2 _<*a*_3 _≤ *l*_0 _- *θ*_*m *_and the values of ⟨*e*_*α*_, *b*_*α'*_, *e*_*α'*_⟩ is

*e*_*α *_- 1 = *j*_*s *_+ *θ*_*m *_+ *a*_1_,   *b*_*α' *_= *j*_*s *_+ *θ*_*m *_+ *a*_2_   and   *e*_*α' *_= *j*_*s *_+ *θ*_*m *_+ *a*_3_.

Similarly, $\left(\begin{array}{c} l_{0}-\theta_{m}\\ 3 \end{array}\right)$ counts all the possible choices of *α' *of length > *θ*_*m *_and of *α *of arbitrary length, the correspondence being *e*_*α *_- 1 = *j*_*s *_+ *a*_1_, *b*_*α' *_= *j*_*s *_+ *a*_2 _and *e*_*α' *_= *j*_*s *_+ *θ*_*m *_+ *a*_3_. Finally, $\left(\begin{array}{c} l_{0}-2\theta_{m}\\ 3 \end{array}\right)$ counts all the possible choices of *α *and *α'*, both of length > *θ*_*m*_. In the cases where such possible choices exist (i.e., if *l*_0 _- 2*θ*_*m *_≥ 3), the correspondence is *e*_*α *_- 1 = *j*_*s *_+ *θ*_*m *_+ *a*_1_, *b*_*α' *_= *j*_*s *_+ *θ*_*m *_+ *a*_2 _and *e*_*α' *_= *j*_*s *_+ 2*θ*_*m *_+ *a*_3_. Then, property **P1 **immediately follows from the inclusion-exclusion argument.   □

## Competing interests

The authors declare that they have no competing interests.

## Authors' contributions

SB, MM, FL and JC drafted the manuscript. FL wrote the proof for the distant segments kernel. SB performed experiments. SB, MM, FL and JC approved the manuscript.

## Supplementary Material

Additional file 1 Source code and data. Web server, classifiers, discriminant vectors and data sets. Click here for file
